# The Therapeutic Potential of Mesenchymal Stromal Cells for Regenerative Medicine: Current Knowledge and Future Understandings

**DOI:** 10.3389/fcell.2021.661532

**Published:** 2021-08-18

**Authors:** Makram Merimi, Rania El-Majzoub, Laurence Lagneaux, Douâa Moussa Agha, Fatima Bouhtit, Nathalie Meuleman, Hassan Fahmi, Philippe Lewalle, Mohammad Fayyad-Kazan, Mehdi Najar

**Affiliations:** ^1^Laboratory of Experimental Hematology, Institut Jules Bordet, Université Libre de Bruxelles (ULB), Bruxelles, Belgium; ^2^LBBES Laboratory, Genetics and Immune-Cell Therapy Unit, Faculty of Sciences, University Mohammed Premier, Oujda, Morocco; ^3^Department of Biomedical Sciences, School of Pharmacy, Lebanese International University, Beirut, Lebanon; ^4^Laboratory of Cancer Biology and Molecular Immunology, Faculty of Sciences-I, Lebanese University, Beirut, Lebanon; ^5^Laboratory of Clinical Cell Therapy, Institut Jules Bordet, Université Libre de Bruxelles (ULB), Brussels, Belgium; ^6^Osteoarthritis Research Unit, University of Montreal Hospital Research Center (CRCHUM), Montreal, QC, Canada; ^7^Department of Natural Sciences, School of Arts and Sciences, Lebanese American University, Beirut, Lebanon

**Keywords:** mesenchymal stromal cells, cell therapy, regenerative medicine, trophic function, immunomodulation, paracrine mechanisms

## Abstract

In recent decades, research on the therapeutic potential of progenitor cells has advanced considerably. Among progenitor cells, mesenchymal stromal cells (MSCs) have attracted significant interest and have proven to be a promising tool for regenerative medicine. MSCs are isolated from various anatomical sites, including bone marrow, adipose tissue, and umbilical cord. Advances in separation, culture, and expansion techniques for MSCs have enabled their large-scale therapeutic application. This progress accompanied by the rapid improvement of transplantation practices has enhanced the utilization of MSCs in regenerative medicine. During tissue healing, MSCs may exhibit several therapeutic functions to support the repair and regeneration of injured tissue. The process underlying these effects likely involves the migration and homing of MSCs, as well as their immunotropic functions. The direct differentiation of MSCs as a cell replacement therapeutic mechanism is discussed. The fate and behavior of MSCs are further regulated by their microenvironment, which may consequently influence their repair potential. A paracrine pathway based on the release of different messengers, including regulatory factors, chemokines, cytokines, growth factors, and nucleic acids that can be secreted or packaged into extracellular vesicles, is also implicated in the therapeutic properties of MSCs. In this review, we will discuss relevant outcomes regarding the properties and roles of MSCs during tissue repair and regeneration. We will critically examine the influence of the local microenvironment, especially immunological and inflammatory signals, as well as the mechanisms underlying these therapeutic effects. Importantly, we will describe the interactions of local progenitor and immune cells with MSCs and their modulation during tissue injury. We will also highlight the crucial role of paracrine pathways, including the role of extracellular vesicles, in this healing process. Moreover, we will discuss the therapeutic potential of MSCs and MSC-derived extracellular vesicles in the treatment of COVID-19 (coronavirus disease 2019) patients. Overall, this review will provide a better understanding of MSC-based therapies as a novel immunoregenerative strategy.

## Graphical Abstract

The road map of MSC review.

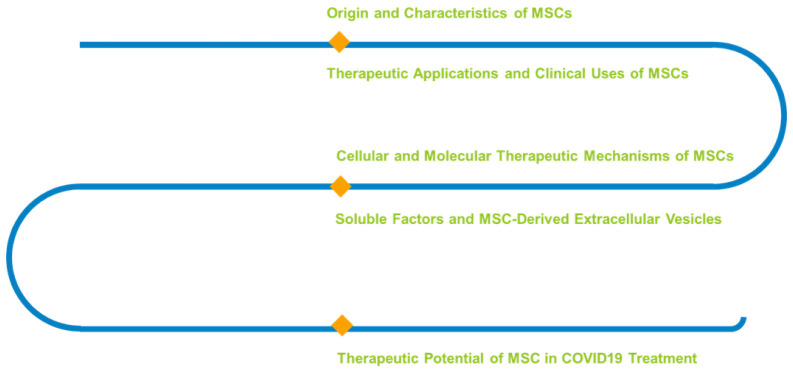

## Introduction

Mesenchymal stromal cells (MSCs) are currently one of the most extensively investigated therapeutic cellular products for clinical applications. MSCs have several characteristics, such as homing to injured tissue sites, immunotropic functions, and paracrine signaling, which allow their use in various conditions, such as tissue regeneration or immunologic/inflammation-related disorders. MSCs were first discovered by Alexander Friedenstein in the late 1960s. They are self-renewable cells with a high ability to proliferate (Bagno et al., 2018). Advances in the techniques for the separation, culture, and expansion of MSCs have enabled their large-scale therapeutic application. This progress accompanied by the rapid improvement of transplantation practices has enhanced the utilization of MSCs in regenerative medicine (Han Y. et al., 2019). This review is organized as follows. Section “Origin and Characteristics of MSCs” discusses the origin and characteristics of MSCs. Section “Therapeutic Applications of MSCs” covers the main therapeutic applications and clinical uses of MSCs, including tissue repair and wound healing, immunomodulatory effects, and diverse therapeutic applications of MSCs. Section “Cellular and Molecular Therapeutic Mechanisms of MSCs” summarizes the cellular and molecular therapeutic mechanisms of MSCs starting from their pleiotropic effects, paracrine action, direct cell–cell contact, and finally mitochondrial transfer. Section “The Secretome of MSCs” presents MSC-derived extracellular vesicles (EVs) as a new therapeutic option and discusses how MSC-secreted EVs also carry several immunomodulatory, antiapoptotic, angiogenic, and antioxidative factors. Section “Outlook on MSCs and MSC-Derived EVs for the Treatment of COVID-19” provides an outlook on the potential therapeutic application of MSCs and MSC-derived EVs in the treatment of coronavirus disease 2019 (COVID-19) patients. Section “Conclusion” presents the conclusion of this review.

## Origin and Characteristics of MSCs

### MSC Discovery

**FIGURE 1:**
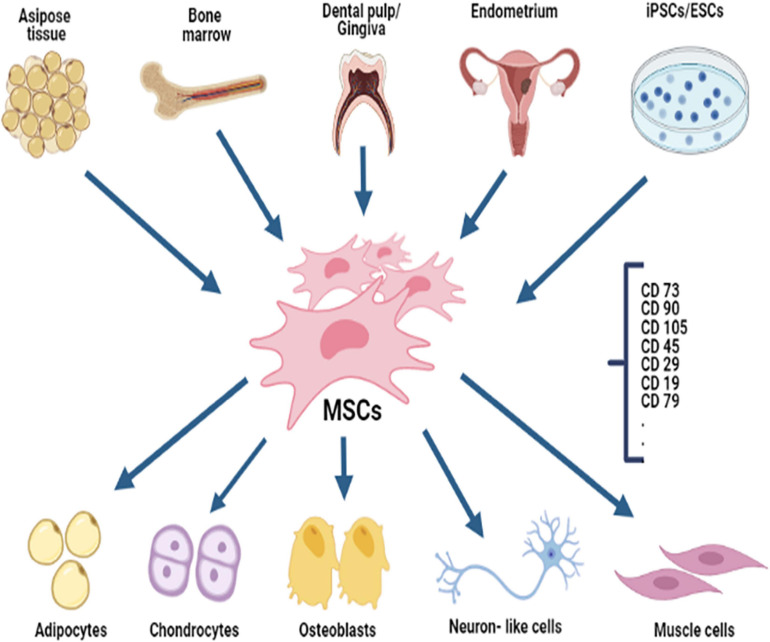
MSCs are isolated from several sources (neonatal, fetal, and adult tissues) and can “in theory” differentiate into different types of cells.

Alexander Friedenstein originally identified colony-forming unit fibroblasts and osteogenic stem cells. Since this time, a number of terms have been used and proposed to describe MSCs. In 1988, Maureen Owen suggested using “stromal stem cells” to indicate that these cells reside in the stromal rather than the hematopoietic compartment (Wilson et al., 2019). Rather than highlighting the cells’ compartmental origin, Arnold Caplan proposed the term “mesenchymal stem cells” in 1991 to emphasize the self-renewal property and differentiation potential of the cells. However, this nomenclature was challenged by James Dennis, who suggested that the cells may be progenitors rather than stem cells. As a result, the term “mesenchymal progenitor cells” was proposed. In 2000, Paolo Bianco and Pamela Gehron Robey coined “skeletal stem cells” to specify that the cells give rise to components of the skeletal system, while only 2 years later, the term “multipotent adult progenitor cells” (MAPCs) was proposed by Yuehua Jiang to describe the multipotent nature and potential progenitor status of the cells (Caplan, 2017). As no direct evidence demonstrated the ability of MSCs to self-renew and differentiate *in vivo*, in 2006, the International Society for Cell and Gene Therapy (ISCT) proposed the term “multipotent mesenchymal stromal cells.” In 2010, Arnold Caplan suggested that the acronym “MSCs” should stand for “medicinal signaling cells” to reflect that the primary therapeutic benefit of MSCs may be attributed to the secretion of bioactive molecules rather than direct cell replacement (Viswanathan et al., 2019). It has been suggested that all multipotent, clonal, and fibroblastoid cells that express MSC markers have a common primary origin, but they adopt different roles during embryogenesis (Figure 1; Brown et al., 2019).

### MSC Product Diversification

More than 50 years of research on MSCs has enabled their isolation from various tissues, such as adipose tissue (Xia et al., 2018), skin, dental pulp, corneal limbus (Shih and Burnouf, 2015), peripheral blood (Tozetti et al., 2017), umbilical cord (UC) tissue (Beeravolu et al., 2017), muscles (Teng et al., 2017), lungs (Pouya et al., 2018), menstrual blood, placental tissues (Macrin et al., 2017; Teng et al., 2017; Aboushady et al., 2018), breast milk, and neonatal tissues (Nakamura et al., 2015; Tozetti et al., 2017). Craniofacial MSCs have high differentiation capability and can rapidly proliferate. As they are easily extracted with minor pain during tooth extraction, craniofacial MSCs may represent another alternative for tissue regeneration, although their specific markers have not yet been well characterized (Zhang et al., 2020).

Although the frequency of MSCs in blood from healthy individuals is extremely low, it may increase under challenging conditions, thus supporting the notion that MSCs can be transiently found “circulating” in blood (Moll et al., 2020a, b). While these diverse sources of MSCs may solve some issues linked to bone marrow (BM), they can display varying levels of highly procoagulant tissue factor and may adversely trigger the instant blood-mediated inflammatory reaction (Witkowski et al., 2016). The former is considered a main trigger for coagulation, whereas the latter has been recognized as a critical threat to graft survival (Moll et al., 2015; Shiratsuki et al., 2015; Christy et al., 2017; George et al., 2018). Moreover, new clinical standards are crucial to complement the minimal criteria for MSC product description (Galipeau et al., 2016; Galipeau and Sensébé, 2018). In this context, Moll et al. (2019) proposed exploring new strategies for screening and monitoring hemocompatibility, as well as developing optimal delivery procedures to guarantee a safe and efficient therapeutic outcome.

Therefore, MSCs can be characterized by the following outstanding properties: (a) can be easily isolated from nearly any tissue, (b) can be differentiated into any cell lineage at its end stage, and (c) can make potential contributions to the management of disease because of their immunological properties (Gao et al., 2016).

### MSC Definition

In 2005, the ISCT determined the minimum benchmark criteria for defining *in vitro* human MSCs: (a) MSCs must be plastic-adherent and display fibroblastoid morphology while preserved in optimal culture conditions; (b) MSCs must present immunophenotypic expression of CD105, CD90, and CD73 and absence of expression of CD34, CD45, CD14, CD19, CD11b, CD79a, and HLA-DR surface indicators; and (c) MSCs must be at least capable of differentiating into osteoblasts, chondroblasts, and adipocytes *in vitro* (Du et al., 2016; Brown et al., 2019). These standards aim to distinguish between mesenchymal stem cells and MSCs, which are not identical. Thus, in addition to their progenitor self-renewal and multilineage differentiation ability, MSCs must possess secretory, homing, and immunomodulatory characteristics (Table 1). Although the basic phenotypic profile must be retained, the International Society for Cellular Therapy (ISCT) committee recommended in 2016 that the following topics be considered: (a) the specific characteristics of each MSC population according to their tissue origin must be determined; (b) the stemness of MSCs should be confirmed *in vivo* and *in vitro*; and (c) robust assays must be implemented to specify the therapeutic action of MSCs (Galipeau et al., 2016).

**TABLE 1:** Criteria to identify MSCs (Dominici et al., 2006).

	Minimum identification criteria	Classification	
1	Adherence to plastic in standard culture condition		
2	Immunophenotypic expression	Positive (≥95%+)	Negative (<2%+)
		CD105	CD45
		CD73	CD34
		CD90	CD14 or CD11b
			CD79 or CD19
			HLA-DR
3	Minimum differentiation *in vitro*	Osteoblasts, chondroblasts, adipocytes	

In the early 1970s, Dexter et al. found that BM-derived MSCs could sustain the growth and viability of hematopoietic cells with growth factor deficiency by secreting trophic factors and cytokines (Han Y. et al., 2019). These findings resulted in significant attention placed on the use of MSCs to repair connective tissue wounds resulting from diseases or trauma. They also introduced the concept of possible regulatory effects on different sides of the immune response (Spees et al., 2016). Despite the similar phenotypes of MSCs, they display heterogeneous biological and functional features. This heterogeneity is due to their different growth and proliferation abilities, multilineage diversity prospects, immunomodulatory potential, and proangiogenic characteristics (Han et al., 2017). For example, higher proliferation rate and less immunogenicity have been reported for MSCs isolated from fetal tissues compared to those obtained from adult BM and adipose (A) tissues. In contrast, placental and BM-MSCs present better proangiogenic competences than MSCs isolated from A and UC tissues (Du et al., 2016).

Although MSCs can be easily differentiated into several end-stage lineages, such as osteogenic, adipogenic, neurogenic, and chondrogenic lineages (Brown et al., 2019; Han Y. et al., 2019), several reasons have hindered their therapeutic application. First, the procedure to obtain MSCs frequently causes pain and discomfort and can lead to donor morbidity. Second, while progressing to the *in vitro* stage, MSC differentiation capability is lessened. Third, the differentiation properties of MSCs are highly affected by environmental factors, such as age, stress, and genetic differences (Mattiucci et al., 2018; Russell et al., 2018). These factors prompted the identification of other favorable sources of MSCs and led to their isolation from the UC and its blood, placenta, and fetal tissues (Beeravolu et al., 2016, 2017). Intravenous infusion is considered the most common route of delivery for various MSC products and has generated a mixed clinical outcome (Ankrum et al., 2014; Galipeau and Sensébé, 2018). BM-MSC infusion proved to be the safest and exclusive source of MSC clinical products until 2008 according to the Food and Drug Administration (Mendicino et al., 2014).

## Therapeutic Applications of MSCs

The trophic and immunomodulatory properties of MSCs have made these cellular products one of the most promising and intensely pursued cellular therapies.

### Tissue Trophic Effect of MSCs

Several properties have made MSCs appealing in the field of regenerative medicine (Hu et al., 2018). Many studies have indicated the ability of MSCs to migrate, engraft, and functionally influence the repair process within the site of injury and damage (Wang Y. et al., 2018; Shojaei et al., 2019). Following injury, anti-inflammatory activities are essential to offset injury, remove dead tissue, and facilitate migration and proliferation of reparative cell types, as well as to increase vascularization and nutrient supply (Toh et al., 2018). In the presence of MSCs, the healing process is accelerated, and the inflammatory reaction is reduced (Li Y. et al., 2019). According to Kim et al. (2019), paracrine signaling and differentiation have both been linked to wound healing process. The potential application of MSCs in tissue repair can take three forms: (1) systemically administered stem cells migrate and home to the injured tissue due to chemical gradients; chemoattraction is mediated by a set of chemokines and their corresponding cell surface receptors; MSCs may migrate to tissues under the action of PDGF, SDF-1 (stromal-derived factor 1), CCL5, CCR2, and CCR3 (Brown et al., 2019); the exact mechanism of stem cell–endothelial interactions at the target site is not well established; however, integrins and selectins facilitate these interactions (Han et al., 2017); (2) differentiation and replacement, in which stem cells engraft and then differentiate into diverse cell types; and (3) the secretion of several factors that influence distinct physiological mechanisms locally and systematically (Fu et al., 2019). It was shown that MSCs release cathelicidin peptide−18, which has an antibacterial effect by slowing down the growth of some bacteria, thus preventing wound infections that impair the healing process (Park et al., 2018). Overall, MSCs promote a proregenerative microenvironment that promotes the tissue local repair and regeneration (Hu et al., 2018). MSCs effectively participate in the tissue repair process through their immunomodulatory, trophic antibacterial, antifibrotic, and proangiogenic functions (Huayllani et al., 2020). MSCs also play a central role during the wound-healing process by coordinating between local cells/progenitors, cytokines, chemokines, and extracellular matrix proteins (Zahorec et al., 2015, Merimi et al., 2021a). Under specific conditions, BM-MSCs may directly or indirectly favor the generation and proliferation of local progenitors, such as endothelial cells and fibroblasts (Hu and Li, 2018; Kucharzewski et al., 2019; Shojaei et al., 2019). The proliferation and functions of keratinocytes, endothelial cells, and fibroblasts are stimulated by molecules present in the secretome of MSCs (Keshtkar et al., 2018). This secretome includes several molecules and cytokines involved in tissue regeneration and immunomodulation (Fu et al., 2019; Huayllani et al., 2020). Several studies have found that the conditioned medium of MSCs (MSC-CM) enhances wound healing and increases the number of dermal fibroblasts and blood vessels and collagen density. MSC-CM enhances the migration and formation of fibroblasts and the presence of several important mediators of wound healing (Pittenger et al., 2019). Several growth factors, such as vascular endothelial growth factor (VEGF) and epidermal growth factor, are released by MSCs, which elevates the recruitment of endogenous cells into the wound. MSCs also control several matrix metalloproteinases (MMPs), such as MMP-1 and MMP-9, which contribute to fibroblast regeneration (Aboushady et al., 2018).

### Immune-Modulating Effect of MSCs

**FIGURE 2:**
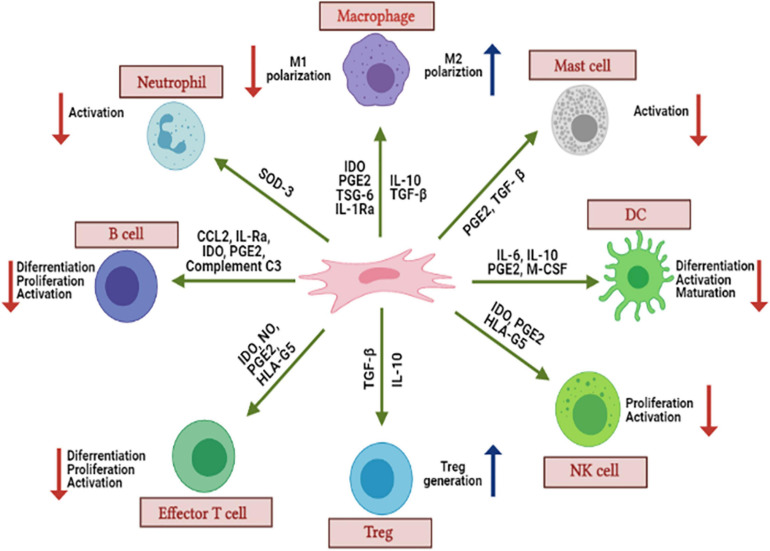
The immunomodulatory effects of MSCs. Various secreted soluble factors (PGE2, TGF-β, HLA-G5, TSG-6, CCL2, IL-1Ra, and IL-10) can activate, suppress, differentiate, and proliferate different immune cell subgroups, including macrophages, mast cells, DC, NK cells, Treg cells, T cells, B cells, and neutrophils. Thus, MCSs will suppress the local inflammation after inhibiting the immune response.

In the context of wound management, MSCs have been acknowledged to have an immunomodulatory effect, which confers them the potential to promote wound repair and decrease inflammation (Praveen Kumar et al., 2019). Because of their immunological features, MSCs play a major role during the tissue repair process (Fu et al., 2019; Praveen Kumar et al., 2019). MSCs display strong immunomodulatory effects mainly mediated by cell–cell contact and secretion of several molecules (Figure 2). These molecules comprise transforming growth factor β (TGF-β), prostaglandin E2 (PGE2), interleukin-10 (IL-10), human leukocyte antigen class I molecule (HLA)-G5, inducible nitric oxide synthase (NOS2), CD39, and CD73 molecules. These factors prevent the proliferation of several immune cells and the secretion of cytokines [IL-1, IL-6, IL-8, IL-12, tumor necrosis factor α (TNF-α), interferon-γ (IFN-γ), TNF-α] and chemokines (CCL2, CCL5) (Vladimirovna et al., 2016; Jiang and Xu, 2020). MSCs inhibit the activation and proliferation of CD4^+^ and CD8^+^ T cells and decrease the production of immunoglobulin by B cells, which makes them appropriate for allogeneic transplantation (Fan et al., 2020). Furthermore, it was demonstrated that MSCs inhibit the allogeneic T lymphocyte response, thus promoting the persistence of skin grafts (Fan et al., 2020). According to Han Y. et al. (2019), MSCs are unlikely to be detected by immune surveillance as they lack significant immune-stimulating antigens (decreased expression of HLA-DR, CD40, and CD86). Thus, they can be adopted in biomedical applications and tissue engineering where no graft rejection after transplantation takes place (Han Y. et al., 2019). MSCs can modulate the function of lymphocytes and macrophages through PGE2 and IL-10 secretion (Hu et al., 2018; Li Y. et al., 2019). On the one hand, PGE2 plays an important role in regulating the shift of T_H_1 cells into T_H_2 cells and thus reduces the activation and proliferation of proinflammatory lymphocytes within the injured tissue (Du et al., 2016). On the other hand, IL-10 contributes to the inhibition of scar formation by preventing the accumulation of collagen I and III and the release of reactive oxygen species (ROS) into the wound area (Honarpardaz et al., 2019). It was suggested that the suppression of allogeneic activated lymphocytes is accompanied by the enhancement of regulatory T (Treg) cells. The inhibition of peripheral monocytes and CD34^+^ progenitor cells from differentiating into antigen presenting cells (APCs), as well as the activation of the cytotoxicity of natural killer (NK) cells, leads to further anti-inflammatory effects. The modulation of the innate and adaptive immune response enables MSCs to suppress fibrosis progression (Ti et al., 2016; Julier et al., 2017; Najar et al., 2019c). Table 2 summarizes many surface markers, secreted proteins, immune-modulating factors, and microRNAs by which MSCs interact with other tissues and cells and may be induced under certain conditions (Merimi et al., 2021b). It has also been proven that chemokines and cytokines that are produced by MSCs contribute to the efficiency and effectiveness of autoimmune disease treatment (Wu Y. et al., 2018).

**TABLE 2:** Markers, factors, and microRNAs to discriminate MSCs (Lv et al., 2014; Camilleri et al., 2016; Oeller et al., 2018; Pittenger et al., 2019; Brinkhof et al., 2020; Jingqiu et al., 2021).

Cluster of differentiation	Growth factors	Regulatory molecules	miRNA
CD9	VEGF	TGF	miRNA-9-5p
CD44	FGF-2	HGF	miRNA-10a
CD54	FLT-3 ligand	PGE2	miRNA-10b
CD58	M-CSF	IL-1RA	miRNA-21
CD62L	G-CSF	IL-6	miRNA-23b
CD71	GM-CSF inducible	IL10	miRNA-24
CD73	SCF	LIF	miRNA-29
CD90	LIF	HLA-G	miRNA-125b
CD105	NFIC	IDO	miRNA-133b
CD106		Inducible NOS	miRNA-143-3p
CD117	**Integrins**	TSG-6	miRNA-145
CDw119	CD49a	Gal-1	miRNA-146b
CD120a	CD49b	Gal-9	miRNA-191-5p
CD120b	CD49c	HO-1	miRNA-199
CD140b	CD49e	LL37	let-7a-5p
CD146	CD51	TGF-β1	miRNA-222-3p
CD166	CD29		miRNA-451
CD221	CD61		miRNA-486-5p
CD222	CD104		miRNA-1224
CD331	CD11a		
CD332	CD18		
CD274	CD49d		
CD276			
CD271			
CD142			
CD36			
CD163			
CD200			
CD273	**Others**		
CD248	YAP		
	WIF1		
CD19	EDIL3		
CD14			
CD34	SSEA-3		
CD45	SSEA-4		
	HLA Class I		
	HLA Class II		
	STRO-1		
	SUSD2		
	MSCA-1		
	CLIC1		
	EPHA2		
	NECTIN2		
	TMEM47		
	GNAI3		
	ALP		

**FIGURE 3:**
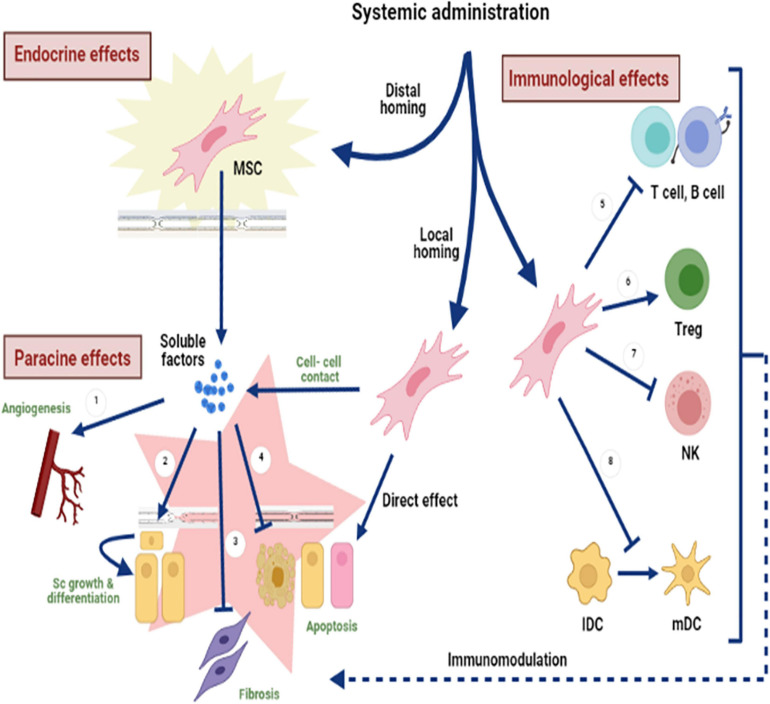
Fundamental mechanisms of MSC-based therapy. These mechanisms differ in their repair activity, depending on various local microenvironments where MSCs can adjust their therapeutic effects accordingly. The systemic administration of MSCs can activate distal (endocrine) or local (paracrine) effects that include cell-mediated actions, which can take different forms, including (1) stimulation of angiogenesis, (2) stem cell growth and differentiation, (3) fibrosis inhibition, (4) apoptosis inhibition, (5) T- and B-cell suppression, (6) initiation of Treg differentiation, (7) NK cell inhibition, and (8) dendritic cell (DC) maturation inhibition.

Upon examining the ability of adipose stem cells (ASCs) to regulate the T_H_17 lymphocyte pathway, a thorough understanding of the biological correlation between T_H_17 lymphocytes and ASCs considering both the cell ratio and the inflammatory environment must be considered (Najar et al., 2019b). Furthermore, it was suggested that the cell ratio and inflammatory primed BM-MSCs significantly affected the production of T_H_17 lymphocytes (Najar et al., 2019a). Zhang Y. et al. (2017) suggested that galectin-1 inhibits the function of DCs by controlling the mitogen-activated protein kinase (MAPK) signaling pathway. MSCs can act either as a suppressor or an enhancer of the immune system by relying on the level of soluble factors in the microenvironment. In this context, Li et al. (2018) demonstrated that when inducible NOS is blocked, MSCs act as immune enhancers by stimulating T-cell proliferation. In contrast, Cuerquis et al. demonstrated that MSCs generate a temporary increase in IFN-γ and IL-2 levels by activating T cells before exerting an immunosuppressive effect (Wang D. et al., 2018). Moreover, MSCs induced with IFN-γ suppressed T-cell proliferation by secreting indoleamine 2,3-dioxygenase (IDO), which catalyzes the conversion of tryptophan to kynurenine. The secretion of programmed death 1 ligand 1 (PD-L1) also contributes to the immunosuppressive effect and thus can be used in the treatment of autoimmune diseases (Figure 3). As such, the microenvironment, especially soluble factor levels along with inflammatory levels, plays an important role in the application of MSC-based therapy (Fan et al., 2020).

### Clinical Indications and Considerations of MSCs

MSCs have been investigated, in both animal and human models, as a therapeutic product to manage various diseases (Harris et al., 2018; Xu, 2018). MSCs are thus indicated for the treatment of degenerative disorders and diseases by displaying antioxidative, antiapoptotic, and immunomodulatory effects (de Witte et al., 2018). Several studies have investigated the potential therapeutic applications of MSCs in Parkinson disease (Hong et al., 2018), multiple sclerosis (Harris et al., 2018; Nasri et al., 2018), degenerative disc disease (Ahn et al., 2015; Beeravolu et al., 2017; Perez-Cruet et al., 2019), Alzheimer disease (Cui et al., 2017; Han et al., 2018), myocardial infarction (MI) (Selvasandran et al., 2018), retinal degenerative disease (Zhang M. et al., 2017; Wang Y. et al., 2018), Crohn disease (CD) (Jahanbazi Jahan-Abad et al., 2018; Brown et al., 2019), and type 1 diabetes mellitus (Evangelista et al., 2018). Moreover, studies have shown that dental MSCs can be used as a complementary source for the regeneration of nerves and have the capability to treat several diseases, such as diabetes, bone deficiency, and neural disorders (Dave and Tomar, 2018).

Several animal models have described a tissue repair capacity following the transplantation of MSCs. In a rat model, BM-MSCs released several mediators, such as fibroblast growth factor 2 (FGF-2), VEGF-1, angiopoietin-2, and TGF-β, which contributed to the healing of MI (Selvasandran et al., 2018). In a mouse model of burn injury, high levels of VEGF and TGF-β1 were suggested to assist burn wound healing by MSCs (Oh et al., 2018). In a rat model, MSCs enhanced fibroblast and keratinocyte differentiation, leading to accelerated wound healing (Xia et al., 2018; Kim et al., 2019). Mouse model of hind limb ischemia revealed that a subset of paracrine factors are efficient biomarkers for predicting vascular regenerative efficacy by Wharton’s jelly-derived MSCs (Kim et al., 2019). In rat periodontal defect model, the implantation of MSC-CM promoted periodontal regeneration by enhancing the mobilization and osteogenesis of local periodontal ligament cells (Kawai et al., 2015). Interestingly, conditioned media (mixed with cosmetic base) from human UC blood-derived MSCs (USC-CM) increased dermal density and decreased skin wrinkle during *in vivo* test with 22 women volunteers (Kim et al., 2018).

The number of registered clinical studies for MSC therapies has exceeded 1,000 worldwide (Moll et al., 2019; Pittenger et al., 2019). Although a meta-analysis of MSC clinical trials has confirmed their safety, the therapeutic efficiency (including the mechanisms of action) of such cellular products formulations should be more scrutinized (Ankrum et al., 2014; Martin et al., 2019). Of all clinical trials using MSCs, the main indications are musculoskeletal diseases with 203 registered studies, 146 trials for central nervous system diseases, 146 trials for immune system diseases, 139 for wounds and injuries, 130 for collagen diseases, 130 for rheumatic diseases, 128 for joint diseases, 127 for arthritis, 127 for vascular diseases, 123 for ischemia, 118 for respiratory tract diseases, 112 for digestive system diseases, and 112 for gastrointestinal diseases. There are 10 globally approved MSC therapies including Alofisel for CD (approved in Europe); Prochymal for GvHD (approved in Canada and New Zealand); Temcell HS injection for graft-vs.-host disease (approved in Japan); Queencell for subcutaneous tissue defects, Cupistem for Crohn fistula, Neuronata-R for amyotrophic lateral sclerosis and Cartistem for knee articular cartilage defects (all approved in South Korea); Stemirac for spinal cord injury (approved in Japan); Stempeucel for critical limb ischemia (approved in India); and Cellgram-AMI for acute MI (approved in South Korea). One of the rare clinical trials in phase III involves the use of allogeneic adipose tissue–derived MSCs for complex perianal fistulas in CD (clinical trial no. NCT01541579). The TiGenix/Takeda phase 3 clinical trial that studied the use of MSCs for complex perianal fistulas in CD is arguably the most successful late-stage MSC trial to date (NCT01541579). Results of this study indicated an effective and safe treatment for perianal fistulas in patients with CD (Panés et al., 2016). Another clinical trial using Alofisel under NCT03706456 is also being actively evaluated for CD management.

Although meta-analysis of clinical trials with first-generation MSC products has demonstrated their safety, their clinical efficiency still needs to be improved. A better understanding of the underlying mechanism of action of MSCs as well as potency assessments pretreatment and posttreatment is key to yield an optimal short- and long-term therapeutic benefit. Therefore, a thorough understanding of patient parameters and complementary treatment protocols are crucial in determining the optimal therapeutic pharmacokinetics (Galipeau and Sensébé, 2018; Aijaz et al., 2019; Hoogduijn and Lombardo, 2019). Efforts should be also developed to improve product design, dosing, and delivery to reach individual clinical needs of patients (Moll et al., 2020b).

### Functionally Improved MSCs by Using Scaffolds

Although stem cells show considerable promise in regenerative medicine, low cell engraftment and survival of the transplanted cells within the target tissue remain key limitation to the successful application of cell-based therapy in the clinic. Indeed, local injection is often associated with poor cell survival and low engraftment due to the harsh and hostile environment at the site of damaged tissue. To ameliorate cell viability/survival and engraftment after injection, stem cells can be combined with biomaterial scaffolds. One of the most widely used biomaterials for the fabrication of scaffolds is hyaluronic acid (HA). HA is the major component of the extracellular matrix of connective tissues (Fraser et al., 1997). It is also abundantly present in UC and synovial and vitreous fluids (Gupta et al., 2019). HA hydrogels can be designed as cell-free therapies through stimulating natural healing processes through the recruitment of endogenous cells (Highley et al., 2016). The combination of HA-based scaffolds and stem cells has been extensively used in cartilage repair. Chung et al. (2014) demonstrated that treatment with a composite of HA and human UC blood-derived mesenchymal stem cells (hUCB-MSCs) led to a superior degree of cartilage regeneration in rat, rabbit (Park et al., 2017), and minipig (Ha et al., 2015) models of disease. Intra-articular injection of a combination of HA and adipose-derived MSCs in a sheep osteoarthritis (OA) model has efficiently blocked OA progression and promoted cartilage regeneration (Feng et al., 2018). Using adult minipig with cartilage defect, the intra-articular injection of MSCs from iliac crest marrow suspended in HA has shown improved cartilage healing both histologically and morphologically (Lee et al., 2007).

Similarly, coadministration of BM-MSCs and HA produced higher regenerative benefit in small and large models of OA, including dogs (Li et al., 2018), and the Hartley guinea pig model of naturally occurring OA (Sato et al., 2012). The combination of HA and stem cells has also been investigated in different models of osteogenesis. The applicability of adipose-derived MSCs and HA showed higher means of bone regeneration in rat model of bone defects (Boeckel et al., 2019). The combination of BM-MSCs and HA successfully indicated bone regeneration in rat calvarial defect model (Kim et al., 2007).

The effect of HA on the therapeutic efficiency of MSCs was further studied in wound healing. Cerqueira et al. (2014) showed that adipose (AD)-MSCs encapsulated within an HA–base hydrogel demonstrated accelerated wound closure, higher re-epithelialization, and neovascularization in a model of skin full-thickness excisional wounds in mice. Comparable results were reported in a separate study using a mouse model of full-thickness (skin) excision wounds in streptozotocin-induced diabetes (da Silva et al., 2017). The results from a clinical trial for safety and proof of concept indicated cartilage regeneration in osteoarthritic patients following the use of a composite of hUCB-MSC. Recently, scaffolds and exosomes from mice BM-MSCs were developed as a combinatorial cell-free system to initiate synergistic tissue immunotrophic effects. Indeed, exosome-laden scaffolds (fibrous polyester materials) proactively facilitated tissue repair in mice skin injury models by favoring M2/T_H_2/Treg responses (Su et al., 2021). Together, all these findings indicate that combined HA and MSCs may constitute an effective strategy in regenerative medicine.

## Cellular and Molecular Therapeutic Mechanisms of MSCs

Two main facets exemplify the therapeutic capabilities of MSCs: the replacement of injured tissue and immunomodulatory activity. The main core mechanism underlying MSC therapy is the pleiotropic effect. This effect allows the release of various soluble factors that display immunomodulatory, antiapoptotic, angiogenic, and antioxidant activities (Figure 3; Fan et al., 2020). The immunosuppressive effect and cell sustainability are regulated by MSCs through cell–cell contact and transfer of mitochondria by tunneling nanotubes (TNTs) to targeted cells (Li H. et al., 2019). Moreover, an anti-inflammatory effect was noted through the release of exosomes, which include numerous microRNAs that enhance cell proliferation throughout tissue regeneration (Huayllani et al., 2020).

### Pleiotropic Therapeutic Effects of MSCs

MSCs play an important role in tissue repair and offer numerous therapeutic applications due to their pleiotropic effects (Hmadcha et al., 2020). Anti-inflammatory and immunoregulatory activities are considered the major pleiotropic contributors to the therapeutic potential of MSCs. Responding to inflammation, MSCs secrete soluble factors, such as TGF-β, TNF-α, IFN-γ, IL-10, and IDO, which alter the inflammatory environment and obstruct the immune system (Kaundal et al., 2018). It was demonstrated that this alteration of immune action triggers a crucial inflammatory mechanism that considerably enhances tissue repair and regeneration by expediting healing and fibrosis (Julier et al., 2017). These pleiotropic effects are also suggested to confer protumor activity to cells. For example, several pivotal studies have shown that MSCs can prevent apoptosis in carcinogenic cells through the release of VEGF and FGF, which are considered soluble prosurvival factors. Numerous studies have agreed on the immunosuppressive effect of MSCs through the secretion of inflammatory factors (Hmadcha et al., 2020). Although MSCs are widely recommended in cell and tissue repair, the engraftment process into the target injured tissue might be influenced by several factors (Lin et al., 2017; Liu et al., 2017). One of these main chemical growth factors is hepatocyte growth factor (HGF), which is a pleiotropic factor that is derived from MSCs. The pleiotropic effect is mediated through enhancing the motility, propagation, and sustainability of cells (Fu et al., 2019). *In vitro*, trafficking of MSCs was linked to significant c-met expression in the presence of HGF concentration gradients. The rat MSC migration process was enhanced through stimulation of the Akt and focal adhesion kinase (FAK) pathways due to the HGF pleiotropic factor (Zhu et al., 2016).

The pleiotropic effect was also mediated by the Abi3bp protein, which acts as an autocrine modulator by significantly enhancing the differentiation of cardiac c-Kit^+^ progenitors (Mori et al., 2018). Moreover, several paracrine factors, such as VEGF, insulin-like growth factor (IGF-1), and FGF, also have pleiotropic characteristics that contribute to the treatment of myocardial injury through different mechanisms. They can affect post–myocardial injury processes such as fibrosis, inflammation, the formation of cardiomyocytes, and neovascularization (Hodgkinson et al., 2016). It has been suggested that directly after MI, the anti-inflammatory reaction is activated through the overexpression of IL-6, which adjusts the paracrine activity of MSCs through the release of VEGF, which enhances the vascularization process. In addition, numerous cytokines, such as IL-1, TNF-α, and IFN-γ, have shown the same inflammatory response as IL-6 through the release of various growth factors that contribute to the regeneration of the myocardium through new capillary formation, cardiomyocyte propagation, and the differentiation of progenitor cells (Bagno et al., 2018). In line with this observation, it was reported that paracrine factors exert pleiotropic actions on repair and regeneration processes through two distinct mechanisms (Hodgkinson et al., 2016). Frizzled-related protein 2 (SFRP2) and hypoxia- and Akt-induced stem cell factor (HASF) are two major paracrine factors that play important roles in cardiac injury by enhancing cardiomyocyte proliferation. While Sfrp2 is linked to the proapoptotic protein Wnt3a in their protective effect, HASF inhibits the death of cardiomyocytes via ε isoform of protein kinase C (PKCε). In addition to their cytoprotective role, SFRP2 inhibits Bmp1 and Sca-1 CPC proliferation, limits fibrosis, and promotes cell differentiation. It was shown that the differentiation process enhanced non-canonical Wnt/planar cell polarity signaling via JNK after Sfrp2 attachment to Wnt6 (Schmeckpeper et al., 2015).

In studying the potential strategies to enhance the therapeutic function of transplanted MSCs in the treatment of damaging neonatal disorders, it was found that the pleiotropic effects are related to paracrine activity and not to regenerative ability. MSCs can detect the microenvironment of the injured area and release various paracrine soluble factors that conduct numerous functions (such as anti-inflammatory, antiapoptotic, antifibrotic, antibacterial, and antioxidant effects) to promote the regeneration and repair of the injured tissue. As such, the efficiency of MSC therapeutic application relies on pleiotropic protection under proper MSC sources, microenvironments, and pharmacokinetics (Park et al., 2018).

### Paracrine Action of MSCs

Although MSCs are widely recommended for cell and tissue repair, the engraftment process into the target injured tissue might be influenced by several factors (Lin et al., 2017; Liu et al., 2017). One of these main chemical growth factors is HGF, which is a pleiotropic factor that is derived from MSCs. The pleiotropic effect is mediated through enhancing the motility, propagation, and sustainability of cells (Fu et al., 2019). *In vitro*, trafficking of MSCs was linked to significant c-met expression in the presence of HGF concentration gradients. The rat MSC migration process was enhanced through stimulation of the Akt and FAK pathways due to the pleiotropic factor HGF (A. Zhu et al., 2016). While investigating the impact of MSCs on myocardial injury, it was reported that paracrine factors exert pleiotropic actions on repair and regeneration processes (Hodgkinson et al., 2016). SFRP2 and HASF are two major paracrine factors acting through two distinct mechanisms that play important roles beyond only a protective one in the case of cardiac injury by enhancing cardiomyocyte proliferation. While Sfrp2 is linked to the proapoptotic protein Wnt3a in their protective effect, HASF inhibits the death of cardiomyocytes via PKCε. In addition to its cytoprotective role, SFRP2 inhibits Bmp1 and Sca-1 CPC proliferation, limits fibrosis, and promotes cell differentiation. It was shown that the differentiation process enhanced non-canonical Wnt/planar cell polarity signaling via JNK after Sfrp2 attachment to Wnt6 (Schmeckpeper et al., 2015).

The pleiotropic effect was also mediated by the Abi3bp protein, which acts as an autocrine modulator by significantly enhancing the differentiation of cardiac c-Kit^+^ progenitors (Mori et al., 2018). Moreover, several paracrine factors, such as VEGF, IGF-1, and FGF, also have pleiotropic characteristics that contribute to the treatment of myocardial injury through different mechanisms. They can affect post–myocardial injury processes such as fibrosis, inflammation, the formation of cardiomyocytes, and neovascularization (Hodgkinson et al., 2016). It has been suggested that directly after MI, the anti-inflammatory reaction is activated through the overexpression of IL-6, which adjusts the paracrine activity of MSCs through the release of VEGF, which enhances the vascularization process. In addition, numerous cytokines, such as IL-1, TNF-α, and IFN-γ, have exhibited the same inflammatory response as IL-6 through the release of various growth factors that contribute to the regeneration of the myocardium through new capillary formation, cardiomyocyte propagation, and the differentiation of progenitor cells (Bagno et al., 2018).

MSCs play an important role in tissue repair and offer numerous therapeutic applications due to their pleiotropic effects (Hmadcha et al., 2020). Their anti-inflammatory and immunoregulatory activities are considered the major pleiotropic contributors to the therapeutic potential of MSCs. Responding to inflammation, MSCs secrete soluble factors such as TGF-β, TNF-α, IFN-γ, IL-10, and IDO, which alter the inflammatory environment and obstruct the immune system (Kaundal et al., 2018). It was demonstrated that this alteration of immune action triggers a crucial inflammatory mechanism that considerably enhances tissue repair and regeneration by expediting healing and fibrosis (Julier et al., 2017). These pleiotropic effects are also suggested to confer protumor activity to cells. For example, several pivotal studies have shown that MSCs can prevent apoptosis in carcinogenic cells through the release of VEGF and FGF, which are considered soluble prosurvival factors. Numerous studies have agreed on the immunosuppressive effect of MSCs through the secretion of inflammatory factors (Hmadcha et al., 2020). In studying potential strategies to enhance the therapeutic function of transplanted MSCs during the treatment of damaging neonatal disorders, it was found that pleiotropic effects are related to paracrine activity and not to regenerative ability. MSCs are able to detect the microenvironment of the injured area and release various paracrine soluble factors that conduct numerous functions to promote the regeneration and repair of the injured tissue, such as anti-inflammatory, antiapoptotic, antifibrotic, antibacterial, and antioxidant effects. As such, the efficiency of MSC therapeutic application relies on pleiotropic protection under proper MSC sources, microenvironments, and pharmacokinetics (Park et al., 2018).

### Direct Cell–Cell Contact

The immunomodulatory effects of MSCs that are applied on the injured sites are either exerted through paracrine mechanisms or via direct cell–cell contact. The cell–cell contact mechanism is crucial for MSCs to stimulate Treg cells and can be adopted for allergic diseases. Furthermore, the immunomodulatory impact of MSCs on T cells and macrophages can be magnified by TSG-6 release through direct cell–cell contact in a proinflammatory environment. Moreover, it was proven that this direct contact decreases the cytotoxicity of NK cells (Gavin et al., 2019). In the context of bone engrafting and cell-based therapeutic applications, MSCs have been differentiated into phenotypes that are similar to pericytes, which promote angiogenesis through direct cell–cell contact (Julier et al., 2017). The interaction with target cells has proven to be one of the key mechanisms in MSC-based therapy. MSCs exert their immunomodulatory effects by promoting Treg cells, inhibiting T cells, and regulating macrophages for numerous inflammatory diseases (Carty et al., 2017). It was proven that T cells are regulated by MSCs through the Fas ligand–Fas relation, B7-H4 molecule, or PD-L1 pathways (Consentius et al., 2015). PD-1 ligand expression, which is present on the MSC membrane, is important for inhibiting the differentiation of allogeneic T_H_17 cells, which depends on direct cell–cell contact. In addition, the inhibition of CD4^+^ and CD8^+^ T-cell propagation occurs via galectin-1 and 3 (Li Y. et al., 2019). A synergy was found between MSCs and Treg cells, where Treg cells promote the release of IDO by MSCs, which in turn inhibits TNF-α and promotes IL-10 in Treg cells. The relationship between MSCs and macrophages cannot be summarized as a simple anti-inflammatory relationship. After direct cell–cell contact, macrophages can phagocytose MSCs and modify their signature to an M2 suppressive phenotype, which clarifies the long-lasting MSC therapeutic effect (Braza et al., 2016). Intriguingly, in some models and under specific conditions, it appears that dead or dying cells or subcellular particles derived from MSCs may contribute to their therapeutic properties. Understanding the necrobiology of MSCs during their therapeutic functions is essential to promote their efficiency and safety (Weiss et al., 2019). Infused MSCs are rapidly phagocytosed by monocytes, which subsequently migrate from the lungs to other body sites. Phagocytosis of MSCs induces phenotypical and functional changes in monocytes, which subsequently modulate cells of the adaptive immune system (de Witte et al., 2018). More specifically, phagocytic clearance of apoptotic MSCs (efferocytosis) by phagocytes is a crucial step in MSC immunosuppression. Efferocytosis could affect the polarization of macrophages and promote M2 anti-inflammatory and regulatory phenotype and function. Such observation may explain how short-lived MSCs mediate therapeutic effects that persist beyond their survival *in vivo* (Ghahremani Piraghaj et al., 2018). This theory is supported by the observation that transfusion of MSCs leads to the prompt phagocytosis of nearly half of lung entrapped MSCs by lung resident macrophages, triggering an IL-10–suppressive efferocytotic response (Galipeau, 2021).

### Mitochondrial Transfer

Mitochondrial transfer has been proposed as one of the original approaches used to restore the respiratory function of injured cells and thus can be adopted in regenerative medicine. This mitochondrial transfer can take different forms, such as microvesicles (MVs), TNTs, gap junctions, and cell fusion mitochondrial transfer (Babenko et al., 2018; Jiang et al., 2019). Mitochondrial transfer from MSCs exerts a protective outcome in the lung, kidney, cornea, bronchoepithelium, and spinal cord (Jiang et al., 2019; Li H. et al., 2019).

## The Secretome of MSCs

Despite being a powerful tool for clinical applications, MSCs have limitations in terms of delivery, safety, and variability of the therapeutic response. Interestingly, the secretome of MSCs was identified as a potential alternative to the cellular product. The secretome is mainly composed of cytokines, chemokines, growth factors, regulatory proteins, and EVs (Eleuteri and Fierabracci, 2019). Despite the similarity in their origin, the secretome of MSCs appears to vary significantly, depending on the age of the donor and tissue sources from which they were isolated. Understanding and profiling the secretome of MSCs will enable the use of the secretome as a new cell-free therapeutic option (Praveen Kumar et al., 2019).

### Extracellular Vesicles

MSC-derived EVs are promising candidates for cell-based and cell-free regenerative medicine, respectively. It has been reported that MSC-derived EVs may be therapeutically more efficient and safer than their cell of origin. EVs have shown stability in circulation, good biocompatibility, and low toxicity and immunogenicity (Shi et al., 2021). These EVs could support the dynamic immunomodulatory activities during tissue repair and regeneration. EVs are likely carriers of lipid, protein, growth factor, cytokines, chemokines, and nucleic acid. They were identified as components of the MSC secretome and propagated the key regenerative and immunoregulatory characteristics of parental MSCs (Wang and Thomsen, 2021). EVs are signaling vehicles in intercellular communication in normal or pathological conditions. EVs convey their functional contents to adjacent cells or distant cells through the circulatory system (Toh et al., 2018). Thus, MSC-derived EVs demonstrate promising cell-free therapy application potential to cure several diseases after monitoring their isolation, dosage, and storage (Zhao et al., 2020). Despite the substantial increase in the number of publications concerning the pathological and physiological properties of EVs, it is still difficult to purify a specific EV population. Such preparations may include heterogeneous exosomes, MVs, microparticles, ectosomes, oncosomes, and other membranous cell–released structures. In view of this, the International Society for Extracellular Vesicles (ISEV) suggested Minimal Information for Studies of Extracellular Vesicles in 2014 (MISEV2014). New guidelines were published in 2018 by the ISEV, which recommends the use of a collective term of EVs unless the biogenesis pathway is demonstrated (Théry et al., 2018). The main objective of MISEV2018 is to develop and improve the EV preparation field; thus, it offers guidelines for proposed protocols to verify specific EV functional activities. Later on, members of four societies (SOCRATES, ISEV, ISCT, and the International Society of Blood Transfusion) proposed to develop new reliable metrics that harmonize the evaluation of the MSC-EV biology and their therapeutic potency. For each EV preparation, the determination of their cell-origin, size, degree of physical and biochemical integrity, composition, and use of a well-characterized MSC-EV biological reference should be performed to guarantee quality and reproducibility (Witwer et al., 2019).

EVs are secreted by numerous cells, including MSCs, where the most important ones are exosomes and MVs. These EVs are crucial in the communication process between cells, where they contribute to both pathological and physiological environments (Konala et al., 2016). Membrane-bound EVs are secreted by somatic cells and contribute to tissue repair, reproduction, and immunomodulatory functions (Lai et al., 2016; Dostert et al., 2017). The main EV markers are CD9, CD44, CD63, CD73, CD80, CD90, and CD105 proteins and antigens; heat-shock protein 60, 70 and 90; and ALG-2–interacting protein X (Li et al., 2018). Microvesicles are produced by various cells through cell membrane budding, which includes cytoskeletal restructuring and depends on the concentration of intercellular calcium (Konala et al., 2016). MVs consist of large quantities of phosphatidylserine proteins, sphingomyelin, ceramide, cholesterol, and CD40 markers. Thus, they contain a load of microRNAs, proteins, and lipids where they bind through receptor–ligand interactions. MVs may either facilitate genetic transmission to the targeted cells, or they may boost angiogenesis by transferring growth factors that will alter the physiological function of the target cell (Merino-González et al., 2016).

It has been recently identified that MVs are the main contributors to tissue regeneration, acting by utilizing biological activity and transmitting information to injured cells (Rani et al., 2015). However, it was recently suggested that MSC exosomes isolated from BM stimulate numerous signaling pathways, mainly STAT3 expression, which participates in its phosphorylation and in the formation of keloid fibroblasts and elevates the expression of growth factors that are mainly related to wound healing, such as IL-8 and C-X-C motif chemokine ligand 1 (CXCL1), nerve growth factor, HGF, IGF-1, and SDF-1 (Shabbir et al., 2015). In the same context, it was demonstrated that STAT3 phosphorylation inhibition reduces the production of collagen in keloid scars. It has been shown that the secretion of exosomes at the wound site plays an immunomodulatory role by preparing a favorable microenvironment through the transfer of microRNAs (Fang et al., 2016; Ti et al., 2016). These exosomal miRNAs inhibit TGF-β2/Smad2 signaling and lessen the development of scars by suppressing myofibroblast construction throughout the wound healing process (Fang et al., 2016).

Recently, MSC-derived EVs have been investigated in numerous clinical applications for their therapeutic potential (Akyurekli et al., 2015). The efficiency of EVs isolated from MSCs efficiency has been associated with their role as antiapoptotic and tubular cell proliferation enhancers in the treatment of acute kidney disease. MSC-derived EVs are involved in the treatment of various neurological diseases, such as Alzheimer disease and multiple sclerosis (Clark et al., 2019; Reza-Zaldivar et al., 2019), by inhibiting the degradation and demyelination of oligodendroglia, which results in motor function progression (Reza-Zaldivar et al., 2019). Moreover, it was demonstrated that MSC-derived EVs have the potential to lessen MI by enhancing angiogenesis, inhibiting apoptosis, supporting proliferation, and regulating the microenvironment. In the context of cartilage repair, MSC-derived EVs have been examined for chondrocyte survival by stimulating matrix formation, preventing apoptosis, and immunomodulatory reactions (Zhang et al., 2018).

EVs that are extracted from MSCs alter the immune system by stimulating Treg cells and the secretion of anti-inflammatory cytokines, controlling macrophages, reducing B lymphocytes, and recruiting neutrophils (Dostert et al., 2017). On the one hand, exosomes enhance the production of monocytes, which differentiate into macrophages through MYD88 (myeloid differentiation gene 88). These macrophages enhance the release of IL-10, which leads to the growth of Treg cells. On the other hand, it was found that macrophage polarization is boosted by miR-146a, turning them to anti-inflammatory ones (Song et al., 2017). Furthermore, the immunosuppressive impact of EVs on B, T, and NK cells, which is facilitated by PD-L1 expression, has been investigated. In addition, galectin-1 and 5′-ectonucleotidase (CD73) exert immunosuppressive effects on T lymphocytes and the production of adenosine, respectively (Del Fattore et al., 2015; Kerkelä et al., 2016). Moreover, miR-16 and miR-100 have been detected and found to exert an antiangiogenic effect in breast cancer by encountering VEGF cells (Pakravan et al., 2017).

In addition, MSC exosomes isolated from the UC have revealed their suppressive function in myofibroblast creation by deterring the TGF-β/SMAD2 pathway and enhancing the presence of some microRNAs, such as miR-21, miR-23a, miR-125b, and miR-145. Consequently, it has been shown that UC exosomes lessen the accumulation of myofibroblasts and scar development (Fang et al., 2016). Moreover, these exosomes have demonstrated an improvement in the re-epithelialization process and cytokeratin 19 and collagen I expression, which contribute to the rejuvenation of skin burns (Zhang et al., 2015a). EVs have an exclusive ability to cross the blood–brain barrier, which contributes to some neurological disorder treatments. This feature is considered superior to traditional MSC-based therapies, which may face some limitations, such as incomplete cell differentiation, immune rejection, malignant alteration, and genetic mutation accompanied by cell transplantation in the treatment of neurological disorders (Li et al., 2018). As such, EVs are considered excellent candidates in regenerative medicine (Fan et al., 2020). Moreover, exosomes that are extracted from MSCs have demonstrated enhanced muscle regeneration by fostering myogenesis, as well as angiogenesis (Nakamura et al., 2015). Several MSC-exosomal microRNAs (miR-19a, miR-22, miR-223) have shown antiapoptotic effects and cardioprotective activity by targeting methyl CpG binding protein 2, transcription 3 (Stat3), and (Mecp2) semaphorin-3A (Sema3A). MSC exosomes can also contribute to renal cell prolongation and growth by enhancing proximal tubular cell sensitivity to IGF-1 by transferring mRNA for the IGF-1 receptor (Zhang et al., 2015a; Song et al., 2017). Finally, MSC-EVs exhibited mixed results in the context of tumor cells. They can act as suppressors or promoters for these cells, depending on their isolation source, stage and type of tumor, and genotype (Lopatina et al., 2016; Whiteside, 2018). As such, EV-based therapy must be cautiously assessed in the treatment of cancer (Fan et al., 2020).

### Antiapoptotic Factors

The inhibition of apoptosis and enhancement of homeostasis can be mediated through the secretion of BCL-2 by MSCs. The elevation of BCL-2 to BAX levels will lead to a decrease in the pathological sensitivity of cells. Moreover, MSCs can produce and release VEGF, HGF, FGF, survivin, IGF-I, stanniocalcin-1 (STC1), and TGF-β, which play similar roles (Ono et al., 2015). In the same context, Zhang et al. indicated that phosphoinositide-3-kinase (PI3K)/Akt contributes to the BCL-2 signaling pathway in terms of antiapoptotic function, thus enabling MSCs to be used in the treatment of ischemia (Zhang Y. et al., 2019). Antiapoptosis activity due to paracrine function under ischemic conditions was exhibited by MSC-conditioned microenvironment where BAX, FAS, TNF receptor, and CASP3 levels are downregulated (Park et al., 2018). In addition to the direct antiapoptotic effect, soluble factors that are secreted and elevated by MSCs, such as VEGF, HGF, FGF, and IGF-I, under hypoxia have been proven to boost cell survival. In particular, VEGF has been proven to upregulate BCL-2 expression, which leads to vascular endothelial cell antiapoptosis, and to stimulate the activating phosphorylation of FAK, which inhibits p53-mediated apoptosis. Therefore, these soluble factors are crucial for cell survival (A. Zhu et al., 2016).

### Angiogenic Factors

Angiogenesis mediates the generation of a new blood vessel network through a complicated process associated with several growth factors, such as HGF, VEGF, and FGF. Numerous studies have demonstrated the ability of MSCs to intensify capillary and blood vessel formation (Merino-González et al., 2016). It has been verified that MSCs exert angiogenic effects that contribute mainly to the regeneration of injured skin, MI, and the treatment of ischemia (Chen et al., 2015; Zhang et al., 2015b). Hung et al. (2007) showed that angiogenesis is stimulated by soluble factors such as monocyte chemotactic protein 1 (MCP-1), IL-6, and VEGF. While MCP-1 is a vital chemoattractant, IL-6 enhances angiogenesis and contributes to the persistence of endothelial cells (Hung et al., 2007). VEGF plays an important role in mediation, migration, and differentiation of endothelial cells through the stimulation of MAPK, PI3K/AKT, and other pathways (Zhu et al., 2018). Moreover, MSCs can enhance angiogenesis through multiple factors, such as SDF-1 and HGF, which facilitate MI repair via SDF-1/C-X-C chemokine receptor type 4 (CXCR4). Additionally, soluble factors in MSCs can be used in the treatment of ischemia because of their angiogenic effects (Zhang Y. et al., 2019).

### Antioxidative Factors

It was demonstrated that there is a significant correlation between ROS levels and chronic diseases such as cancer, immune disorders, and neurological diseases (Kreuz and Fischle, 2016). MSCs, through the secretion of STC1, can decrease apoptosis induced by ROS and regulate oxidation reduction. STC1 inhibits angiotensin II–enhanced superoxide formation in cardiomyocytes and stimulates uncoupling proteins 2 and 3 (UCP2 and UCP3), which promote mitochondrial respiration and alveolar epithelial cell persistence (Ono et al., 2015). Moreover, it was shown that STC1 suppresses the NLRP3 inflammasome, which lowers the release of mitochondrial ROS. Furthermore, Chen et al. (2010) verified that HO-1 enhances the paracrine effect, which decreases inflammation and oxidation induced by LPS. As such, MSCs are capable of secreting numerous antioxidative factors in different microenvironments (Fan et al., 2020).

## Outlook on MSCs and MSC-Derived EVs for the Treatment of COVID-19

The emergence of severe acute respiratory syndrome coronavirus 2 (SARS-CoV-2) is the cause of a global pandemic present in more than 150 countries and has highlighted the multifactorial and complex syndrome named sepsis (Wu et al., 2020). SARS-CoV-2 enters host cells via the cell surface angiotensin-converting enzyme 2 receptor present on many cells, such as alveolar type 2 and blood vessel cells (Hoffmann et al., 2020). In approximately 20% of patients, SARS-CoV-2 leads to an excessive and aberrant host immune response, resulting in severe lung disease characterized by acute respiratory distress syndrome (ARDS) and multiorgan dysfunction. In COVID-19 patients, the immune system produces large amounts of inflammatory factors (IL-2, IL-6, IL-7, MCP-1, TNF-α, etc.), causing a cytokine storm responsible for ARDS, organ failure, and secondary infections (Mehta et al., 2020). Several therapeutics are being evaluated, and because of their anti-inflammatory and immunomodulatory properties, allogeneic MSC therapy has been proposed. The ISCT and ISEV recognized the therapeutic potential of MSCs and their derived EVs as treatments for COVID-19. Efforts should be focused on the generation of appropriate manufacturing and quality control provisions, preclinical safety and efficacy data, rational clinical trial design, and proper regulatory oversight (Börger et al., 2020).

In line, several preclinical studies have reported the protective effect of MSCs in sepsis murine models and septic shock (Laroye et al., 2017). Recent studies have evaluated the efficiency of MSCs for ARDS treatment. A phase I trial reported good tolerance and the absence of major adverse effects (Zheng et al., 2014; Wilson et al., 2015). The START study (phase IIa) compared a single intravenous dose of cryopreserved BM-MSCs with placebo in patients with moderate to severe ARDS and reported a significant improvement in oxygenation in the MSC group but without improvement in survival (Matthay et al., 2019). A single-center prospective randomized Russian clinical trial of BM-MSCs in neutropenic patients with septic shock reported hemodynamic stabilization, vasopressor withdrawal, attenuation of respiratory failure, and shortening of the neutropenia duration period (Galstyan et al., 2018). A preliminary analysis of a phase 1 and 2 study using a good manufacturing practice product of allogeneic BM-derived MAPCs in ARDS (MUST-ARDS) demonstrated improvement of oxygenation, reduced lung edema, and decreased proinflammatory cytokines (Bellingan et al., 2019). Two reports from China have shown initial results from MSC therapy in COVID-19 patients. Compassionate use of UC-MSCs (three doses) in a 65-year-old patient requiring mechanical ventilation and with multiple organ failures led to clinical improvement in vital signs and the cessation of mechanical ventilation after the second dose (Liang et al., 2020). A second study reported the use of MSCs from undefined sources to treat seven patients with ARDS. All patients showed clinical improvement after 2 days and remarkable improvements in inflammation markers and in the immune cell repertoire (Leng et al., 2020). Many other clinical trials utilizing MSCs have been initiated for the treatment of COVID-19 (>80 studies declared on the clinical trial.gov website). Most of the trials use allogeneic MSCs, predominantly BM- and UC-MSCs, and perform repeated infusions. Interestingly, few trials use MSC-CM or EVs able to exert similar functions to MSCs (Sengupta et al., 2020).

By exerting their immunomodulatory effects, MSCs may induce tissue repair and organ protection for patients with a confirmed infection. While the need for MSC-based therapy in COVID-19 is apparent, integrating both preclinical and clinical strategies into the current guidelines is critical for safe and effective therapies (Moll et al., 2020a). Future randomized controlled trials are also needed to confirm the therapeutic potential of MSCs to treat COVID-19 patients.

## Conclusion

MSCs have generated significant interest over the past decade as a novel therapeutic strategy for a variety of diseases. In this review, we discussed the therapeutic properties of MSCs during tissue repair and regeneration. MSCs interact and modulate the local progenitor and immune cells that are involved in tissue homeostasis. Moreover, several immunological and inflammatory signals may critically influence the effects and properties of MSCs. It is essential to understand the impact of the tissue environment on the fate and functions of MSCs. Understanding the paracrine pathway involved in the healing process governed by MSCs is also important to obtain efficient and safe regenerative medicine applications.

## Author Contributions

MM and MN conceived and designed the review. All authors listed have made a substantial, direct and intellectual contribution to the work and contributed to manuscript writing, revision, reading, and approval of the submitted version.

## Conflict of Interest

The authors declare that the research was conducted in the absence of any commercial or financial relationships that could be construed as a potential conflict of interest.

## Publisher’s Note

All claims expressed in this article are solely those of the authors and do not necessarily represent those of their affiliated organizations, or those of the publisher, the editors and the reviewers. Any product that may be evaluated in this article, or claim that may be made by its manufacturer, is not guaranteed or endorsed by the publisher.
